# Surveillance based estimation of burden of malaria in India, 2015–2016

**DOI:** 10.1186/s12936-020-03223-7

**Published:** 2020-04-16

**Authors:** Ashwani Kumar, Himanshu K. Chaturvedi, Ajeet Kumar Mohanty, Surya Kant Sharma, Mantoshkumar S. Malhotra, Arvind Pandey

**Affiliations:** 1 Indian Council of Medical Research, National Institute of Malaria Research, Field Unit, Campal, Panaji, 403 001 Goa India; 2 Indian Council of Medical Research, National Institute of Medical Statistics, Ansari Nagar, Medical Enclave, New Delhi, 110 029 India; 3 Indian Council of Medical Research, National Institute of Malaria Research, Sector 8, Dwarka, 110 077 New Delhi India

**Keywords:** Malaria burden, API, Estimation of cases, Deaths, Incidence, Prevalence, Test positivity rate

## Abstract

**Background:**

India has launched the malaria elimination initiative in February 2016. Studies suggest that estimates of malaria are useful to rationalize interventions and track their impact. Hence, a national study was launched to estimate burden of malaria in India in 2015.

**Methods:**

For sampling, all 624 districts of India were grouped in three Annual Parasite Incidence (cases per thousand population) categories, < two (low); two-five (moderate) and > five (high) API. Using probability proportional to size (PPS) method, two districts from each stratum were selected covering randomly 200,000 persons per district. Active surveillance was strengthened with 40 trained workers per study district. Data on malaria cases and deaths was collated from all health care providers i.e. pathological laboratories, private practitioners and hospitals in private and public health sectors and was used for analysis and burden estimation.

**Results:**

Out of 1215,114 population under surveillance, 198,612 (16.3%) tests were performed and 19,386 (9.7%) malaria cases were detected. The malaria cases estimated in India were 3875,078 (95% confidence interval 3792,018–3958,137) with API of 3.05 (2.99–3.12) including 2789,483 (2740,577–2838,389) *Plasmodium falciparum* with Annual Falciparum Incidence of 2.2 (2.16–2.24). Out of 8025 deaths investigated, 102 (1.27%) were attributed to malaria. The estimated deaths in India were 29,341 (23,354–35,327) including 19,067 (13,665–24,470) confirmed and 10,274 (7694–12,853) suspected deaths in 2015–2016.

**Conclusions:**

Estimated malaria incidence was about four folds greater than one million reported by the national programme, but three folds lesser than thirteen million estimated by the World Health Organization (WHO). However, the estimated deaths were 93 folds more than average 313 deaths reported by the national malaria programme in 2015–2016. The 29,341 deaths were comparable with 24,000 deaths in 2015 and 22,786 deaths in 2016 estimated by the WHO for India. These malaria estimates can serve as a benchmark for tracking the success of malaria elimination campaign in India.

## Background

The World Health Organization (WHO) has reported 22% decline in malaria from the estimated 271 (177–382) million cases in the year 2000 to 212 (144–294) million in 2015 [1]. The reduction in estimated malaria attributable mortality is even more impressive from 856,728 (594,760–1204,220) deaths down to 426,791 (218,780–630,698). With these trends, the WHO has advocated elimination of malaria in at least 35 countries by the year 2030 [2]. Following the WHO path, India has launched the malaria elimination initiative in 2016.

The first set of global disease burden modelling studies was carried out a couple of decades ago for estimation of communicable and non-communicable diseases, injuries and deaths [3, 4]. Many studies have also been conducted using country data and subjecting it to different methodologies, assumptions and epidemiological models to generate estimates of malaria burden [1, 5–11]. However, wide gaps between the estimates and the reported incidence have been the subject of intense debate calling to question not only deficiencies in surveillance and reporting systems, but also methodologies adopted to arrive at such estimates.

Outside of Africa, India is the main contributor to malaria related morbidity and mortality in the South-East Asia. Hence, several attempts have been made to estimate malaria burden in India from time to time using secondary data [1, 8, 12]. Mortality estimates for the year 2002 were provided by Dhingra et al. based on cause of death by verbal autopsy (COD VA) data of the Million Death Study from 2001 to 2003 [13, 14]. It was estimated that below the age of 70 years, there were 205,000 deaths attributable to malaria/annum; < 5 years of age-55,000, 5–14 years of age-30,000 and 15–69 years of age-120,000 deaths. As the death estimates were about 300 times greater than the deaths reported by the Indian national programme, this publication triggered intense debate on the methodology adopted. It was surmised that besides issues related to the time gap between the death and the verbal autopsy, the overlapping of symptoms of other diseases with malaria could have influenced responses of the respondents [15, 16]. Further, based on Vital Registration System and Medical Certification of Cause of Death, Kumar et al. estimated about 146,000 and 141,000 deaths due to malaria in India respectively in 1997 and 1998 [17]. A committee constituted by the Government of India arrived at an estimate of 9.751 million cases and 40,297 deaths due to malaria (30,014–48,660) in the year 2010 [18].

More recent global malaria mortality trends published suggest 46,970 (14,757–94,945) deaths for individuals of all ages in India in 2010 [5]. These included 4826 deaths (781–14,437) in children less than 5 years of age and 42,145 (11,340–88,615) deaths for individual of 5 years and older. Curiously, malaria ranked 7th among 291 causes of death and injuries in both 1990 and 2010 [9].

Malaria burden estimates at national and sub-national levels are vital not only as benchmark for priority setting and resource allocation but also to gauge programmatic achievements during disease elimination process. As malaria burden estimates based on a nationally representative sample of primary morbidity and mortality data are lacking, the present study is first such attempt globally which was carried out in three different malaria-endemic zones representing India in 2015–2016.

## Methods

### Sampling frame and sample size

A national sampling frame was prepared based on data provided by National Vector Borne Diseases Control Programme (NVBDCP) for stratification and selection of the clusters as a basic requirement of a sampling design. A list of all 624 districts of India with annual parasite incidence (API) of last 3 years (2011–2013), which was obtained from NVBDCP, served as the sampling frame. Based on the maximum API of last 3 years, all the districts were divided into three strata (S1, S2 and S3) of endemicity, i.e., high (S1: API ≥ 5), moderate (S2:2 ≥ API < 5) and low (S3: API < 2).

### Selection of study districts

**Fig. 1:**
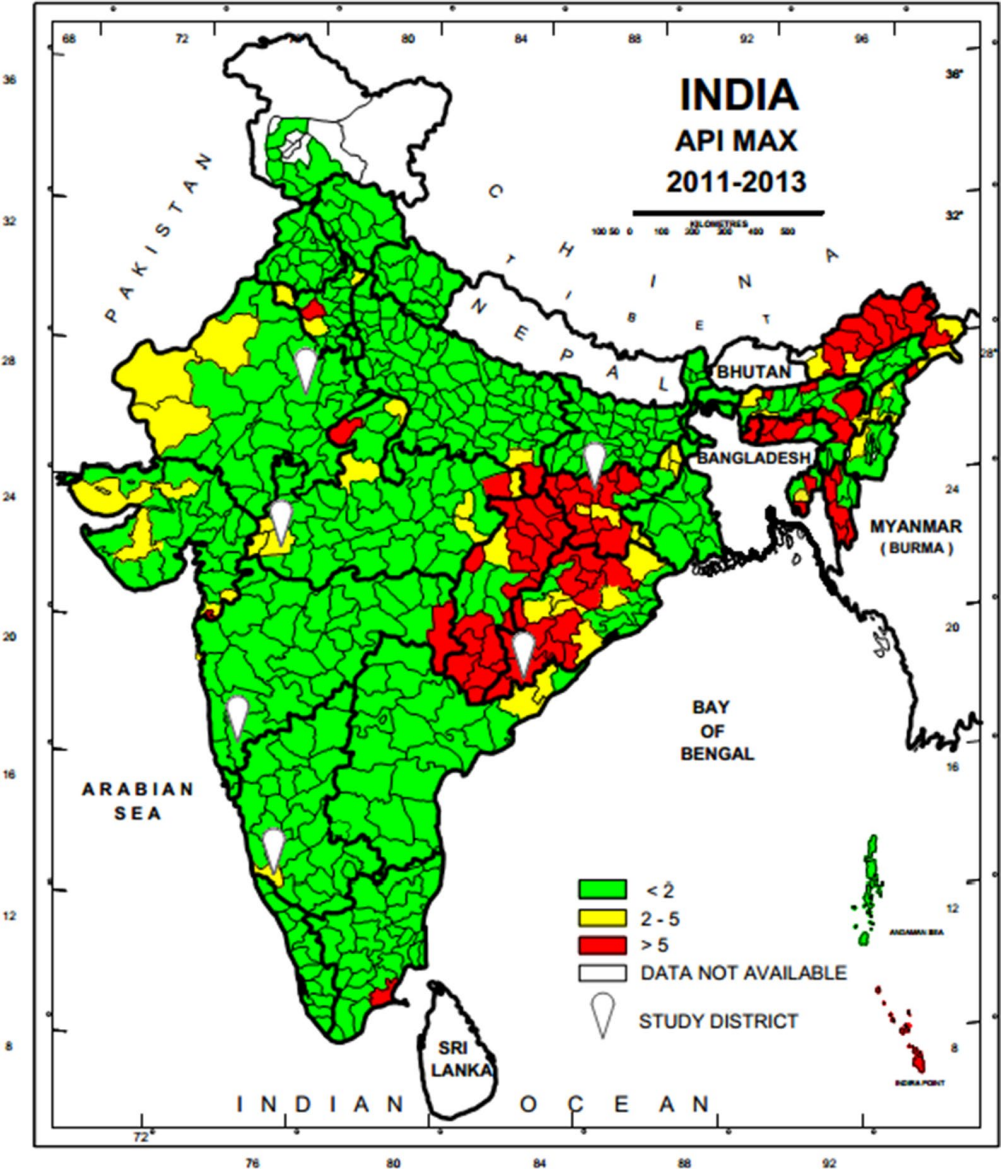
Map of India showing geographical location of six study districts (white balloons) for capturing malaria morbidity and mortality. Firstly district level stratification of India was done on the basis of three Annual Parasite Incidence (API) classes, < 2, 2–5 and > 5 taking into consideration APImax of malaria from 2011–2013 and then two study districts from each of the three strata were randomly selected as per PPS sampling method to conduct malaria burden estimation study

The sample size was worked out to provide the reliable estimate of API for each region and death rate due to malaria at the national level. It was based on the median API of the malaria endemic strata (7/1000 in S1, 3/1000 in S2 and 0.5/1000 in S3) with 10% margin of error in S1 and S2 and 20% margin of error in S3, 95% confidence interval, 10% non-response and design effect-2. As the median API of low endemic region (districts with API < 2) was low, the computed required sample size worked out to 400,000 persons. The same sample size was uniformly applied to the other two regions to maximize the possibility of capturing both malaria and death cases. The total sample size was thus 1200,000 from all the three regions. In this manner, two representative districts each from low, moderate and high burden districts and overall 6 study districts were selected in the country (Fig. 1). Further, three Primary Health Centres (PHCs) were selected randomly from the list of all PHCs of each selected district so that study population size within each selected PHC was approximately 70,000. In case the selected PHC was smaller (i.e. population was < 60,000), some population of the adjacent PHC or a sub-Centre was included in the study area to obtain the desired sample size. Similarly, the larger PHC (i.e. with a population > 80 000) was divided to select a contiguous segment of required population size. Overall study population of surveillance area was about 0.2 million/district. For concurrent death enumeration, an adjacent PHC of similar size and epidemiological features matching the surveillance PHC area was also selected.

### Study personnel

Six Technical Assistants (one in each district), 18 Field Workers (three in each district) and 240 (40 in each district) Voluntary Surveillance Monitors (VSMs), a statistical Assistant, an Epidemiologist, Consultant Biostatistician and 6 Data Entry Operators along with Co-Is and PIs were engaged for managing the project activities. The VSMs were chosen from the study or neighbouring villages/wards where they were assigned surveillance work. Field staff was trained in performing Rapid Diagnostic Test (RDT), preparing blood smears, filling up of study forms and record keeping. The VSMs worked in close collaboration with village Accredited Social Health Activists (ASHAs) and Multi-Purpose Workers (MPWs) and were instrumental in finding fever cases and testing their blood for malaria followed by treatment of malaria cases.

### Surveillance

#### Active surveillance

The community level active surveillance was carried out in each selected villages of PHC areas in rural and municipal areas or ward in urban area. House listing and complete enumeration of the study population was carried out in each study area (PHCs/Municipal wards) before initiation of surveillance by the 12 to 15 VSMs with help of ASHAs. One VSM was assigned a population of about 5000 or 1000 houses (one or more villages of PHCs or section of municipal areas) fortnightly to carry out the active surveillance of fever cases for the period of 12 months. During the surveillance, all the fever cases were recorded by the VSMs and their blood test for malaria was performed by the surveillance team. They were also referred to the PHCs or Sub-centres for confirmation of malaria and treatment. All the fever cases identified during the active surveillance were recorded with the result of blood test in the active surveillance format (A) and compiled at the end of every month. Field Workers (FW) were supervising VSMs, solving day to day problems, cross-checking all the cases reported by the VSMs every month, and maintaining the supplies of study related material and carrying out of the verbal autopsy of each death case using prescribed formats (D). Technical Assistants (TA) were responsible for overall field activities, logistics, and coordination with state health officials, solving of local problems, supervision, data collation and reporting to the project co-investigators.

#### Passive surveillance

**Fig. 2:**
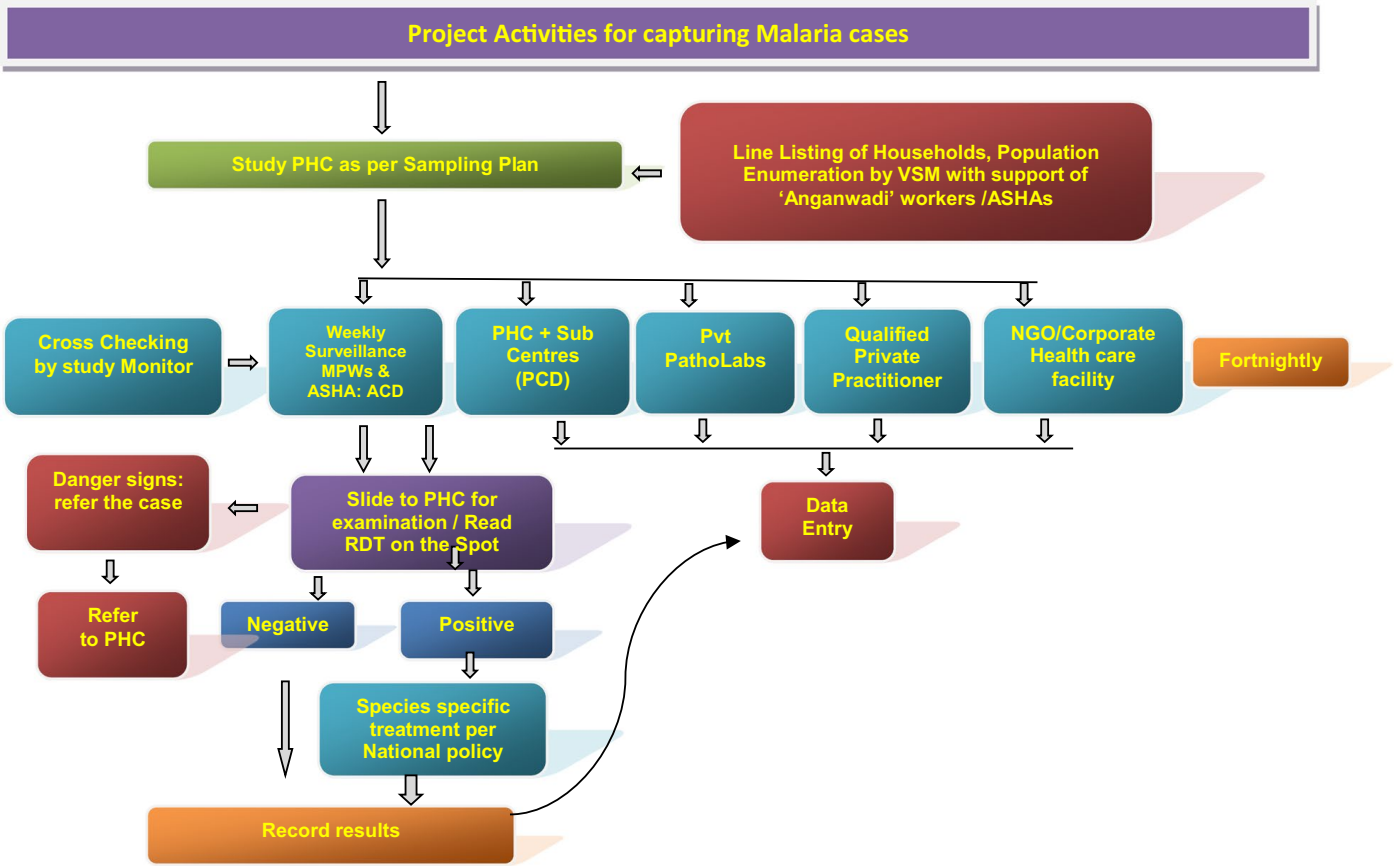
Flow diagram of activities carried out in the surveillance areas of the study districts to capture malaria cases by instituting surveillance and from enlisted health facilities, diagnostic laboratories, private practitioners and institutions

**Fig. 3:**
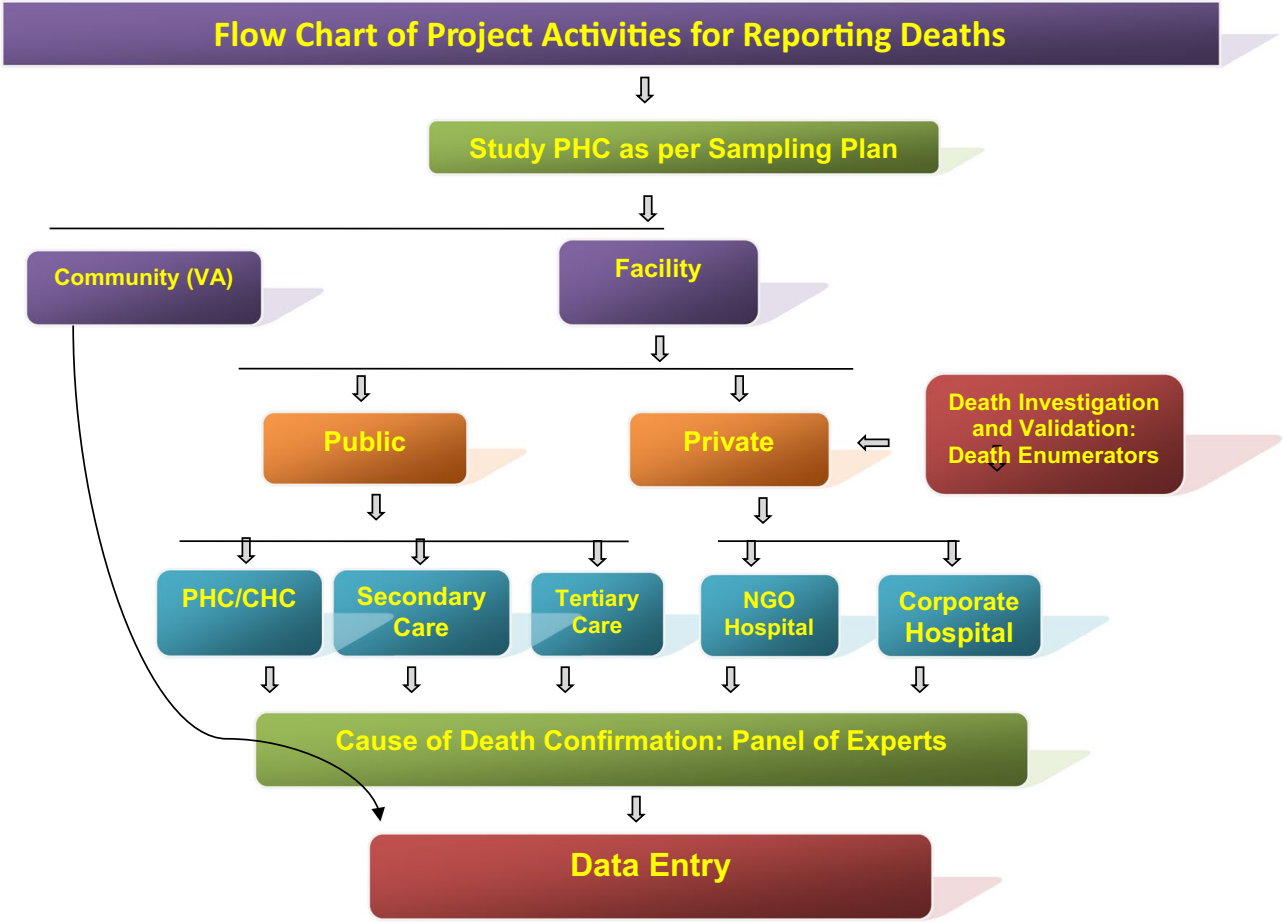
Flow diagram of the surveillance activities carried out in the study areas of the study districts to capture death cases by eliciting information from ‘Panchayats’ (Local self-Government bodies in the study villages), Municipal Councils/corporations, burial/cremation ground, public, community leaders, schools, shops, ASHAs, hospitals, etc. Verbal Autopsy of death cases was done after a fortnight of death occurrence by visiting residence of the deceased and information was captured on standard VA instrument in local language. Each VA report was examined by two medical experts independently for labeling the cause of death viz., probably due to malaria, confirmed due to malaria, cause other than malaria and unclassified death (cause cannot be discerned)

The information related to passive cases detection was collated to ascertain morbidity and mortality due to malaria in the study PHC areas in rural areas and municipal wards in the urban areas (Figs. 2 and 3). All government and private health facilities in the study area and the vicinity were listed and empanelled to capture malaria cases coming from the study surveillance area and accessing these facilities. All records of fever and malaria cases were cross checked in both active and passive lists to avoid duplication. Blood tests were performed by bivalent RDTs (for both *Plasmodium vivax* and *Plasmodium falciparum*) and by making thin and thick blood smear of fever cases encountered in the study population. Treatment of confirmed malaria cases was done by study personnel following the current national anti-malarial drug policy [19]. Data of all fever cases and their blood tests results collected through active and passive surveillance and also the VA of death cases were finally checked with name and address for confirmation of cases belonging to the surveillance study population by the project team (VSMs, FW, TA) during the district level monthly meeting and finally confirmed by the project co-investigator before sending the data to the central team for analysis.

### Deaths and cause of death assignment

Information on deaths captured through different sources (including hospital, death registry, cremation/burial records) was recorded. A pre-designed and tested verbal autopsy (VA) tool was filled up for all the death cases by visiting household of the deceased on day 15 post death. Two independent physicians after auditing all VA forms assigned the cause of death. In case of disagreement between the two physicians, a third physician was consulted and final cause of death based on agreement between any two physicians was assigned. If available, the medical records related to the death cases were also taken into consideration for cause of death assignment.

### Data analysis

Data collected during the surveillance period of 1 year was analysed to obtain the crude and weighted estimates of annual incidence rates, death rates. The weights were calculated according to the study design adjusting the differences in sample coverage in each study area of district and overall estimates were obtained using the population weight of three endemicity strata (S1–S3).

## Results

### Malaria morbidity

**Fig. 4:**
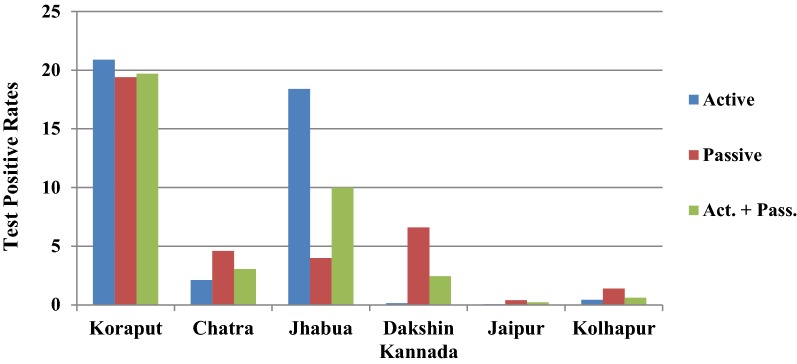
Test Positive Rates for malaria showed high variaility in active and passive collections in six study districts

**Fig. 5:**
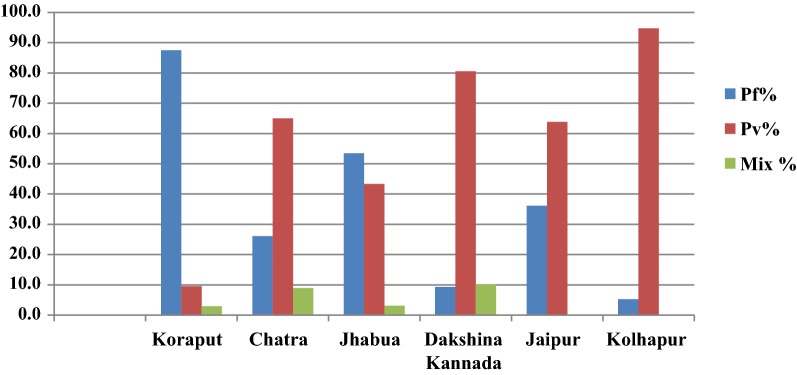
The proportion of *P. falciparum* and *P. vivax* varied in the 6 study districts

In Koraput district with high malaria endemicity, 15,563 cases with test positivity rate (TPR) of 19.69% and in Chatra district 916 cases (TPR 3.06%) were detected from study population. In the moderate endemic districts, malaria cases were 1947 with TPR of 10.01% in Jhabua, but in Dakshin Kannada 791 cases (TPR: 2.45%) were captured. In the low endemic areas, 36 and 133 malaria cases were captured in Jaipur and Kolhapur districts respectively with < 1 TPR (Fig. 4, Table 1). Koraput and Jhabua districts showed predominance of *P. falciparum* (55–88%), while remaining 4 districts viz., Chatra, Dakshin Kannada, Jaipur and Kolhapur, showed predominance of *P. vivax* (44–95%). Mixed infections were reported in all the districts except Jaipur and Kolhapur which had extremely low incidence (Fig. 5). The observed Annual Parasite Incidence (API) which denotes malaria cases per 1000 population in Koraput being 74.5, was two-four folds higher than reported API in the previous 3 years, but observed API was within the range of reported API in Chatra, Dakshin Kannada and Jaipur districts. However, it was higher (9.3) in Jhabua compared to 3.6–8.0 reported in the previous years. In Kolhapur, though API of 0.7 was low in general, yet it was 17–35 folds higher when compared with reported API (0.02–0.04) in the years 2012–2014 (Table 1).

**Table 1:** District wise total malaria cases including active and passive cases captured during the study period one year (2015–16)

Districts	Study population	Total BSE/RDT	Total positive	*P. f*	*P. v*	Mix (*P. v *+* P. f*)	TPR	Pf%	API: 2015–2016	API range: 2012–2014
Koraput^#^	208,981	79,021	15,563	13,618	1493	452	19.69	87.5	74.5	17.6–37.2
Chatra^$^	199,364	29,971	916	239	595	82	3.06	26.1	4.6	3.0–7.7
Jhabua^$^	208,395	19,456	1947	1042	844	61	10.01	53.5	9.3	3.6–8.0
Dakshina Kannada^$^	201,292	32,265	791	74	637	80	2.45	9.4	3.9	2.5–3.71
Jaipur^$^	204,849	16,144	36	13	23	0	0.22	36.1	0.2	0.05–0.29
Kolhapur^*^	196,460	21,755	133	7	126	0	0.61	5.3	0.7	0.02–0.04

BSE, blood slides examined; RDT, rapid diagnostic test; *Pf, Plasmodium falciparum*; *Pv, Plasmodium vivax*; TPR, test positive rate

Study period: ^#^Sept. 2015-Aug. 2016; ^$^Aug. 2015-July 2016; *July 2015-June 2016

### Malaria attributable deaths

**Fig. 6:**
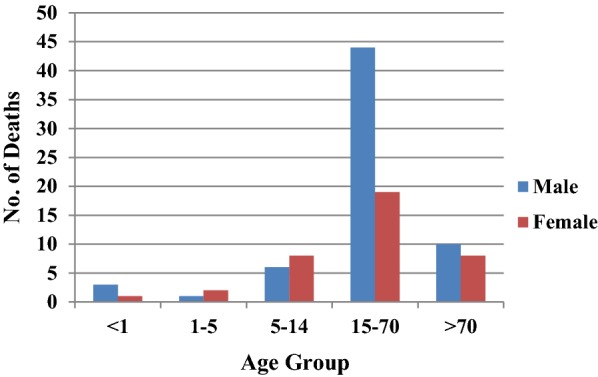
Age and sex distribution of deaths in the study districts

From the six study districts, 8025 deaths were investigated (Table 2). Ten physicians assigned cause of death based on verbal autopsy (VA) narratives and available medical records. In high-malaria endemic Koraput district, out of 946 verbal autopsies performed, 60 and 35 deaths were labelled as attributed to malaria by the medical experts as confirmed and suspected deaths, respectively. In Chatra district only two deaths, one confirmed and one suspected were caused by malaria. In moderately malaria-endemic region, one suspected death due to *P. falciparum* malaria in Jhabua district and three malaria deaths (all due to *P. vivax*) in Dakshin Kannada district were captured from the hospital records. In low malaria-endemic region, only one confirmed death due to complicated *P. falciparum* was reported in Kolhapur district (Table 2). Of these 102 total malaria deaths, 65 (63.7%) were among males and the rest 37 (36.3%) were among females in a male: female ratio of 1.75:1. The number of deaths was greater among males as compared to females in all the age groups except in children 1–14 years of age (Fig. 6). The number of deaths (62) was greatest in the broad age group of 15–70 years. In this age group, deaths were twice greater in males than in females. However, 18 deaths occurred among persons over 70 years of age involving both sexes almost equally.

**Table 2:** Deaths reported from the study districts in death and fever surveillance arms and cause of death assigned by medical experts

District	Area/ arm	Study popn.	Verbal autopsy done (N)*	No. of confirmed malaria death N (%)	No. of suspected malaria deaths N (%)	No. of death-causes other than malaria N (%)	No. of un-classified deaths N (%)
Koraput^#^	Death	199,822	946	60 (6.34)	35 (3.69)	832 (87.9)	19 (2.0)
Chatra^#^	Death	210,200	756	1(0.1)	1(0.1)	736 (97.3)	18 (2.4)
Jhabua	Death	201,143	558	0	1(0.17)	557 (99.8)	0
	Surveil.	208,389	403	0	0	379 (94.0)	24 (6.0)
Dakshin Kannada	Death	202,000	1143	0	0	823 (72.0)	320 (28.0)
	Surveil.	201,292	950	3 (0.31)	0	657 (69.1)	290 (30.5)
Kolhapur	Death	210,970	1006	0	0	729 (72.4)	277 (27.5)
	Surveil.	196,460	964	1 (0.1)	0	696 (72.2)	267 (27.7)
Jaipur	Death	201,227	640	0	0	616 (96.2)	24 (3.75)
	Surveil.	200,622	659	0	0	648 (98.3)	11 (1.7)

^*^The number of deaths captured varied in study districts as community response was uneven. ^#^In Koraput and Chatra tough terrain and poor accessibility permitted collation of death information verbal autopsy to the Death area/arm only

### *Plasmodium falciparum* prevalence and mortality rates

When computed *P. falciparum* prevalence rate (*Pf*PR) was highest (17.8%) in Koraput district followed by Jhabua (5.66%). In the remaining 4 districts, *Pf*PR was low from 0.03 to 1.0% (Table 3). *Plasmodium falciparum* specific mortality rates (*Pf*MR) showed wide variation. In high malaria-endemic district, *Pf*MR was 0.67% and 0.62% respectively in Koraput and Chatra, while in Jhabua district *Pf*MR was low at 0.09% and in Dakshin Kannada district it was nil as all 3 deaths were due to *P. vivax*. Incidentally, in Kolhapur district with only 7 *P. falciparum* cases, one *P. falciparum*-attributable death was confirmed and hence and *Pf*MR rate stood abnormally high at 14.2%. The crude death rate was 47.5/100,000 persons in case of Koraput but < 1 in the remaining districts and the overall rate was 5.01/100,000 persons (Table 3).

**Table 3:** Shows *Plasmodium falciparum* prevalence rate (*Pf*PR), *P. falciparum* specific mortality rate (*Pf*MR) and Crude malaria death rate (malaria deaths/100,000 popln.)

District	Study popn. (a)	BSE (b)	*Pf* + mixed (*P. f *+* P. v*) cases (c)	*Pf*PR (%) (c)*100/(b)	Malaria deaths [conf. + sus.] (d)	*Pf*MR (%) (d)*100/(c)	Crude malaria death rate/100,000 popln. (d)*100,000/(a)
Koraput	199,822	79,021	14,070	17.8	95	0.67	47.5
Chatra	210,200	29,971	321	1.07	2	0.62	0.95
Jhabua	409,532	19,456	1103	5.66	1	0.09	0.24
Dakshin Kannada	403,292	32,265	154	0.47	3^#^	0	0.74
Jaipur	407,430	16,144	13	0.08	0	0	0
Kolhapur	401,849	21,755	7	0.03	1	14.2^	0.25
Total	2,032,125	198,612	15,668	7.88	102	0.63^$^	5.01

*Pf*PR, *Plasmodium falciparum* Prevalence Rate; *Pf*MR, *Plasmodium falciparum* Specific Mortality Rate

^#^All 3 deaths due to *P. vivax;*^$^Excludes *P. vivax* deaths in Dakshin Kannada district; ^sample size small (n = 7)

### Estimation of malaria morbidity and mortality burden

Based on the sample data of 6 districts, the number of malaria cases and deaths attributable to malaria were estimated for India by the weighted estimates of various rates such as annual fever rates and annual incidence of malaria and death rates due to malaria (Additional files 1, 2, 3, 4: Table S1–S4). As per Expert Group of Population Projection of India report, the projected population of India as on March 1, 2016 (mid of study period 2015–16) was worked out as 1.268 billion which was used to arrive at population-based malaria morbidity and mortality estimates. The population share of the three strata to the total population of India was 5.2% in case of high, 8.4% in moderate and 86.4% in case of low malaria endemic districts in strata S1, S2 and S3 respectively. This categorization was as per the initial sampling frame prepared for this study design.

The estimated Annual Parasite Incidence (API) was 41.66 for high, 6.53 for moderate and 0.39 for low malaria endemic areas (Table 4). Overall, the weighted estimate of API for the country worked out to 3.05 (2.99–3.12) per thousand population. Based on weighted estimates of API and standard error, the estimated number of malaria cases in the country ranged from 3792,018 to 3958,137 during the study period of one year with point estimate of 3875,078 malaria cases in India (Table 4).

**Table 4:** Estimated total and *P. falciparum* malaria cases in India based on API and AFI found in high, moderate and low endemic strata

Area	Popn. in 2016 (billion)	API (crude)	API (estimated)	AFI (crude)	AFI (estimated)	Estimated Pf & mixed cases (*P. f *+ *P. v*)	Estimated total cases
High	0.06	40.38	41.66	35.27	36.57	2412,973	2749,139
Mod.	0.107	6.68	6.53	3.07	3.00	320,324	696,415
Low	1.096	0.43	0.39	0.05	0.05	56,186	429,524
Total (India)	1.269		3.05 (CI: 2.99–3.12)		2.2 (CI: 2.16–2.24)	2789,483 (CI: 2740,577–2838,389)	3875,078 (CI: 3792,018–3958,137)

API, Annual Parasite Incidence; AFI, Annual Falciparum Incidence; *Pf, Plasmodium falciparum;* UI, uncertainty interval

The estimated AFI (Annual Falciparum Incidence) based on *P*. *f* malaria cases (both *P. f* & Mix *P. f *+ *P. v*) for the three regions was 36.57 for high, 3.0 for medium and 0.05 for low endemic area (Table 4). Overall, the weighted estimate of AFI for the country was 2.20 (2.16–2.24) per thousand populations. Based on weighted estimates of AFI and standard error of estimate, the estimated number of *Pf* malaria cases in the country was worked out between 2740,577 and 2838,389 with point estimate of 2789,483 *P. falciparum* cases (including mix infections).

### Estimation of deaths due to malaria

All deaths attributed to malaria were categorized as confirmed and suspected deaths. In high malaria-endemic areas, the death rate due to confirmed malaria was estimated at 25.44/100,000 population and death rate due to suspected malaria as 15.0/100,000 population with overall death rate due to malaria as 40.44/100,000 population (Table 5). In high malaria prevalence area which embodies a population of 0.066 billion, the estimated deaths due to confirmed malaria were 16,789 and estimated deaths due to suspected malaria were 9901 hence 26,690 total deaths. In moderate malaria prevalence area, the death rate due to confirmed malaria was 0.5368/100,000 of population and death rate due to suspected malaria was 0.34/100,000. Hence in moderate prevalence area with 0.107 billion population, the estimated deaths were 945 of which 572 were due to confirmed malaria and 373 were due to suspected malaria. In low malaria burden area, the death rate due to confirmed malaria was 0.1556/100,000 and death rate due to suspected malaria was nil. In low malaria prevalence areas of India which had 1.096 billion populations, the estimated deaths due to confirmed malaria were 1706 and suspected malaria deaths were nil. Hence, the overall point-estimates of deaths due to confirmed malaria were 19,067 (13,665–24,470) and the point estimate of deaths due to suspected malaria was 10,274 (7694–12,853) with total deaths of 29,341 (23,354–35,327) due to malaria in a population of 1.269 billion in India (Table 5).

**Table 5:** Estimated deaths due to malaria in India based on results of Verbal Autopsy and death information from health facilities in six study districts

Area	Population in 2016 (billion)	Deaths in 400,000 (SRS 2016)	Estimated death rate^$^ (per 100,000 population)			Est. malaria deaths^$^		
			Confirmed malaria	Suspected malaria	Confirmed + suspected	Confirmed	Suspected	Confirmed + suspected
High	0.06	2760	25.4431	15.0044	40.4474	16,789	9901	26,690
Mod	0.107	2920	0.5368	0.3495	0.8863	572	373	945
Low	1.096	2480	0.1556	0	0.1556	1706	0	1706
Total (india)	1.269	–	1.5026	0.8096	2.3122	19,067 0(13,665–24,470)	10,274 (7694–12,853)	29,340 (23,354–35,327)

^$^weighted

## Discussion

The foregoing effort on estimation of morbidity and mortality attributable to malaria has been made by conducting surveillance based prospective study for the first time in India. The findings of the study confirm that malaria scenario is highly diverse in the country. The observed malaria incidence in high endemic Koraput district situated in Odisha state of India was two-four folds greater than expected. On the other hand, Jhabua which represented districts of the country with moderate incidence showed much higher incidence of malaria than reported in the earlier years (Table 1). In the remaining districts, however, the observed incidence was quite as expected with over all indication that the sample districts could capture wide spectrum of variability of malaria normally observed in India. The same was also true for *P. falciparum* and *P. vivax* variability found between the study districts (Fig. 5). The overall contribution of *P. falciparum* and *P. vivax* was 72% and 28%, respectively in the present study as opposed to two-third and one-third ratio reported by the national programme, showing a differential of about 5% in both parasite species in 2015 [20].

The total number of estimated cases of malaria were about four folds more than about 1 million reported to the National Malaria Control Programme of India in the years 2015–2016 [20]. However, they were about one-third of 13 million (9.9–18 million) estimated by WHO for India for the year 2015 and 2016 [1, 23]. Interestingly, 71% of the incident cases were contributed by only 4.7% of the total 1.268 billion population spread across eastern and north-eastern states of India. These states also contributed to 86.5% of the total estimated *P. falciparum* cases (Table 4).

During the present study, 93% of the reported deaths were in Koraput district of Odisha, a state which is highly malaria endemic with predominance of *P. falciparum*. It may be mentioned that Odisha state with only about 4% (43.7 million) population contributed 42% to the total reported malaria cases and 55.1% to the total *P. falciparum* cases in the country. Odisha also contributed to 31.8% of the total reported deaths due to malaria in India in 2015 [20]. The age-gender composition of deaths as seen in Fig. 6 confirms previously reported trends [5, 12].

Malaria deaths in children in south and south-east Asia have been steadily decreasing since 1980 and accounted for a small proportion of the global deaths in this age group in 2010 [5]. Many studies have suggested that adult mortality due to malaria in India far exceeds in proportion than earlier known [5, 12, 13]. Even in Africa, adult absolute mortality is greater than child mortality than it was previously believed [23]. This has implications on the distribution of resources among affected populations both for surveillance as well as for vector control. Accordingly, the intervention focus needs to be widened covering both children as well as adults. This has epidemiological significance too. The re-enforced immunity after repeated infections in lower ages, which is expected to reduce adult malaria mortality, is short lived or not strong enough to prevent complications and deaths in adults in India [23].

India contributed 6% to the global estimated malaria cases and 49% to *P. vivax* cases in the year 2015 [1]. The country also contributed 6% to total deaths estimated and 51% to *P. vivax* mortality figures [1]. The time trends of malaria mortality estimated for India and endemic countries for 1980–2010 have also been recently published [5]. The WHO has reported a decline in the estimated number of malaria cases in India by 38% from 21 million in 2010 to 13 million in 2015 and by 55% (9.59 million) in 2017 and malaria deaths by 27%, i.e., from 33 000 in 2010 to 24 000 in 2015 and by 50% (16,733) in 2017 [1, 24]. It is pertinent to mention that in the last decade, various programmatic changes have been introduced in India viz., improvement in the health infrastructure under National Health Mission and health care delivery over time through 600,000 village level health workers known as Accredited Social Health Activists (ASHAs). These workers have been providing better on-the-spot diagnosis (with RDT) of malaria at the doorsteps of the local people and enhanced ACT (artemisinin-based combination therapy) access for the treatment of *P. falciparum* malaria besides decentralized procurement and improving supply chain of LLINs to the communities in malaria high risk areas, etc. All these factors must have impacted trends of malaria morbidity and mortality in the country as observed in this study.

The estimated 29,341 (23,354–35,327) deaths from the primary data were comparable with WHO estimates of 24,000 (1500–47,000) for the year 2015; 22,786 (1580–45,300) for the year 2016 and 16,733 (1200–31,900) for the year 2017 [1, 24]. However, these estimates were significantly lesser than 46,970 (14,757–94,945) estimated for India by Murray et al. and 205,000 by Dhingra et al. [5, 13] while the deaths estimated in the present study were 76 folds greater than 384 deaths reported in India in 2015 [20].

The well cited limitations of the VA, notwithstanding, the observed crude *P. falciparum* mortality rate of 0.63% was on expected lines [21, 22]. Najera and Hempel have reported that outside of Africa, malaria mortality has been estimated to be 1% of the estimated *P. falciparum* malaria incidence [23]. In the present study, malaria mortality rate was similarly 1.05% which was estimated taking 29,341 deaths as numerator and 2789,483 estimated *P. falciparum* cases as denominator in this study. Further, this agreement in *P. falciparum* mortality rates of the current study with that of earlier studies suggests that the methodology adopted in the current study for the burden estimation was quite appropriate. The recall period during VA was kept the shortest possible as 15 days during the study to elicit accurate information from the respondents. Another caveat of the study is that study population was enumerated just before initiation of surveillance and all the households and individuals were listed for follow up, but there is no information recorded about their movement or lost to follow up during the surveillance period.

Most importantly, this study has provided estimates of malaria cases and deaths in India at a time when they are most needed, i.e., at the inception of the malaria elimination campaign in the country. For such a vast and diverse country as India, the investigators recommend estimation of malaria burden at suitable intervals during the entire phase of malaria elimination till the target year 2027 and possibly beyond. This will offer distinct advantages as (1) the progress towards malaria elimination could be tracked when the current annual incidence is compared with the baseline numbers of malaria cases and deaths in different strata; (2) in the pre- and post-elimination phases, if any setbacks are observed in the targets, they could be timely addressed and (3) the regional priorities for resource allocation could be appropriately set to address residual transmission when the country is approaching malaria elimination targets to accelerate transmission control efforts and prevention of resumption of active transmission of malaria in areas of the country where malaria is eliminated.

## Conclusions

The incidence of malaria in India were estimated at about 4 million in the year 2015–16. The estimates were four-fold improved over the number of malaria cases reported by the National Malaria Control Programme. Though they were about the three-fold lower than those which were estimated by the WHO for India for the same year, but the present estimates were based on an active surveillance sample survey design. The estimates depicted that over 70% of the total incident cases of malaria and 87% of the *falciparum* malaria cases were from about 5% of India’s population spread over eastern and north-eastern states of India.

The survey, during the year, estimated 29,341 (23,354–35,327) deaths due to malaria. They were the improved estimates over the earlier estimates and were comparable to the estimates provided by the WHO for the same period. The present estimates can serve as the benchmark for tracking the success of malaria elimination campaign in India.

## Supplementary information

**Additional file 1: Table** **S1.** Estimated Fever Rate in high, moderate and low malaria strata.

**Additional file 2: Table** **S2**. Estimated Fever Rate for all the study districts.

**Additional file 3: Table** **S3.** Estimated Test Positive Rate Crude).

**Additional file 4: Table** **S4.** Estimated Malaria Mortality Rate (weighted).

## Data Availability

All the data and documents supporting the results of this study have been archived in ICMR-National Institute of Malaria Research, Dwarka, New Delhi. The raw and analysed data are available with ICMR-National Institute of Medical Statistics, Ansari Nagar, New Delhi.

## Abbreviations

API: Annual parasite incidence
AFI: Annual falciparum incidence
PPS: Probability proportion to size
WHO: World Health Organization
COD: Cause of death
VA: Verbal autopsy
NVBDCP: National Vector Borne Diseases Control Programme
VSM: Voluntary Surveillance Monitor
RDT: Rapid diagnostic test
ASHA: Acredited Social Health Activist
MPW: Multipurpose health worker
TPR: Test positive rate
PfPR: *Plasmodium falciparum* prevalence rate
PfMR: *Plasmodium falciparum* mortality rate
