# Supplementary material for: Surveillance based estimation of burden of malaria in India, 2015–2016
Source: Malar J. 2020 Apr 16;19:156. doi: 10.1186/s12936-020-03223-7 (PMC7160962; doi:10.1186/s12936-020-03223-7)
Supplement: Supplementary file 1 — Additional file 1: Table S1. Estimated Fever Rate in high, moderate and low malaria strata. [file 12936_2020_3223_MOESM1_ESM.docx]

***Table S1:* Estimated Fever Rate in high, moderate and low malaria strata**

| **Area** | | **Study Pop.** | | **BSE** | | **Fever Rate (%)** | | **Est. Fever Rate* (%)** |
| --- | --- | --- | --- | --- | --- | --- | --- | --- |
| **High** | 408345 | | 108992 | | 26.69 | | 26.79 | |
| **Mod.** | 409687 | | 51721 | | 12.62 | | 12.60 | |
| **Low** | 397082 | | 37899 | | 9.54 | | 9.67 | |
| **Total (India)** | 1215114 | | 198612 | | 16.35 | | 10.81 | |
| *Rates are estimated by using weights which were developed according to the population proportion of three malaria endemicity areas and the study design; BSE= Blood Smears Examined | | | | | | | | |
