# Supplementary material for: Surveillance based estimation of burden of malaria in India, 2015–2016
Source: Malar J. 2020 Apr 16;19:156. doi: 10.1186/s12936-020-03223-7 (PMC7160962; doi:10.1186/s12936-020-03223-7)
Supplement: Supplementary file 2 — Additional file 2: Table S2. Estimated Fever Rate for all the study districts. [file 12936_2020_3223_MOESM2_ESM.docx]

***Table S2:* Estimated Fever Rate for all the study districts**

| **District** | **Study Pop.** | **BSE** | **Fever Rate (Crude)** | **Fever Rate**^*^ **(Estimated)** |
| --- | --- | --- | --- | --- |
| **Koraput** | 208981 | 79021 | 37.81 | 38.56 |
| **Chatra** | 199364 | 29971 | 15.03 | 15.02 |
| **Jhabua** | 208395 | 19456 | 9.34 | 9.33 |
| **Dakshina Kannada** | 201292 | 32265 | 16.03 | 15.88 |
| **Kolhapur** | 196460 | 21755 | 11.07 | 11.27 |
| **Jaipur** | 200622 | 16144 | 8.05 | 8.07 |
| **Total (India)** | 1215114 | 198612 | 16.35 | 10.81 |
| *Rates are estimated by using weights which were developed according to the population proportion of three malaria endemicity areas and the study design. | | | | |
