# Supplementary material for: Surveillance based estimation of burden of malaria in India, 2015–2016
Source: Malar J. 2020 Apr 16;19:156. doi: 10.1186/s12936-020-03223-7 (PMC7160962; doi:10.1186/s12936-020-03223-7)
Supplement: Supplementary file 3 — Additional file 3: Table S3. Estimated Test Positive Rate Crude). [file 12936_2020_3223_MOESM3_ESM.docx]

***Table S3:* Estimated Test Positive Rate Crude)**

| **Area** | **BSE** | **Pos.** | **TPR (Crude)** | **TPR*(Estimated)** |
| --- | --- | --- | --- | --- |
| **High** | 108992 | 16479 | 15.12 | 15.55 |
| **Mod.** | 51721 | 2738 | 5.29 | 5.18 |
| **Low** | 37899 | 169 | 0.45 | 0.41 |
| **Total (India)** | 198612 | 19386 | 9.76 | 2.83 |

*Rates are estimated by using weights which were developed according to the population proportion of three malaria endemicity areas and the study design; TPR= Test Positive Rate irrespective of type of test used (smear or rapid test).
