# Supplementary material for: Surveillance based estimation of burden of malaria in India, 2015–2016
Source: Malar J. 2020 Apr 16;19:156. doi: 10.1186/s12936-020-03223-7 (PMC7160962; doi:10.1186/s12936-020-03223-7)
Supplement: Supplementary file 4 — Additional file 4: Table S4. Estimated Malaria Mortality Rate (weighted). [file 12936_2020_3223_MOESM4_ESM.docx]

***Table S4:* Estimated Malaria Mortality Rate (weighted)**

| **Area** | **Estimated Cases** | **Est. Deaths*** | **Est.**  **Case Fatality Rate*** |
| --- | --- | --- | --- |
| **High** | 2749139 | 16789 | 0.61 |
| **Mod.** | 696415 | 572 | 0.08 |
| **Low** | 429524 | 1706 | 0.40 |
| **Total (India)** | 3875078 | 19067 | 0.49 |

*Based on deaths attributable to malaria infection confirmed by microscopy/RDT
